# Coronavirus: Where Has All the Health Economics Gone?

**DOI:** 10.34172/ijhpm.2020.108

**Published:** 2020-06-22

**Authors:** Cam Donaldson, Craig Mitton

**Affiliations:** ^1^Yunus Centre for Social Business & Health, Glasgow Caledonian University, Glasgow, UK.; ^2^Centre for Clinical Epidemiology and Evaluation, Vancouver Coastal Health Research Institute, University of British Columbia, Vancouver, BC, Canada.

**Keywords:** Health Economics, Trade-Offs, Opportunity Cost, Economic Appraisal, Coronavirus

## Abstract

As the coronavirus disease 2019 (COVID-19) pandemic continues to unfold there is an untold number of trade-offs being made in every country around the globe. The experience in the United Kingdom and Canada to date has not seen much uptake of health economics methods. We provide some thoughts on how this could take place, specifically in three areas. Firstly, this can involve understanding the impact of lockdown policies on national productivity. Secondly, there is great importance in studying trade-offs with respect to enhancing health system capacity and the impact of the mix of private-public financing. Finally, there are key trade-offs that will continue to be made both in terms of access to testing and ventilators which would benefit greatly from economic appraisal. In short, health economics could – and we would argue most certainly should – play a much more prominent role in policy-making as it relates to the current as well as future pandemics.

## Introduction

As debates unfold about how coronavirus and coronavirus disease 2019 (COVID-19) have challenged evidence-based medicine,^1^ the same might apply to health economics. Apart from being seen as a branch of disease modelling, which itself has been recognised as an inexact science,^2^ health economics seems to have been absent from important debates based on its lifeblood of resource scarcity, and consequent trade-offs, each of which have been laid bare in the current crisis. This seems even more contradictory when two of the basic structures of society – health and the economy – are each threatened, but with the discipline connecting the two – health economics – not represented at top tables of advice.^3-8^ Even more, given the undoubted world-leading recognition of health economics in some of the worst-affected countries.

Health economists may not have been welcome, or thought of, at the outset of this crisis. When throwing everything you have at a major existential problem, it is not good to have a branch of the ‘dismal science’ sitting in the room saying “Wait a minute….Hold on…?” But, with public health leaders and other clinical experts having done their best to flatten the curve, it is timely to reflect on what health economics might offer in advance of, within and in emerging from such pandemics. This is about health economics in its broadest sense; as much a branch of moral philosophy, laying out issues arising from trade-offs being made, as a quantifier of costs and benefits of specific costs and benefits from such trade-offs. So, what might these trade-offs be, and what approach might health economists take to thinking about and analysing them?

### Health Versus the Economy? Only “at the Margin”

‘Guns versus butter’ has been invoked on many occasions by politicians in the past to show the large-scale trade-offs that societies have to make in times of crisis; usually in wartime. COVID-19 has brought this large-scale type of question to light in terms of health versus the economy. This may seem straightforward at first sight. What might be the benefits gained – in lives saved, years of life saved or quality adjusted life years saved – from the increased healthcare expenditures and the social and economic support packages thrown at COVID-19 by various governments? This should include assessment of the opportunity cost of patients whose procedures and treatments were curtailed. However, also of great importance, and less straightforward, is examining first the fall in national productivity that will ensue – and has already ensued – from lockdown policies, and second, as healthcare spending is directly linked to gross domestic product, future economic downturn resulting in spending less on healthcare and social care and other key support services in years to come, were this to transpire. This should be possible to model.^9^

Such models are likely to emerge after the fact, and could lead to health economists being accused of explaining what happened when it is too late but having had little to say as the crisis unfolds; as was largely the case with our parent discipline of economics back in 2008. But there is a clear opportunity for the use of economics, through ‘marginal analysis’ - that is, in considering trade-offs across health and the economy as we go. Currently, governments are presenting the easing of lockdown as a win-win, ie, easing lockdown for low-risk economic activities as long as there is no threat to the R-value of disease spread. However, in short order, genuine trade-offs will emerge which will result in us posing questions such as “how much might we allow the R-value and the death toll to rise for gains in economic activity?” In other words, and inescapably, what is the value of life? Estimates of such values exist for use in policy-making,^10,11^ and it is a question to which economists could contribute through further work on public preferences and on modelling values revealed by the policy choices made at the margin in this particular context. It is clear that trade-offs and choice making are at the very heart of health economics. If one is to rely only on (at times unfounded) epidemiological models, it is impossible to assess the value of a given set of actions (ie, costs relative to benefits) and thus the decision-maker does not possess adequate information to determine how best to allocate limited societal resources.

On the evening news, we started by seeing Finance Ministers providing updates on aid packages and the economy as a whole while Health Ministers independently have given the latest COVID-19 case counts. Models are possibly being constructed in the background, but do those for the economy link back across to health, and *vice versa*, in ways that a health economist might suggest? Now, we are moving towards the trade-offs outlined, and it appears, at least in both the United kingdom and Canada, to be happening without drawing on the health economics methods at hand.

### Health for the Economy: Preparedness, Capacity and Public-Private Mix

This year, the *LSE-Lancet Commission of the Future of the NHS* (National Health Service) was due to report against a backdrop considerable strain on health and social care resources.^12^ COVID-19 has raised the stakes even further, especially in light of the economic outlook. Nevertheless, if not instantly, economies will recover, and longer-term questions of preparedness of health and social care systems post-COVID-19 will remain. More specifically, these are about capacity and public-private mix.

With respect to capacity, South Korea and Germany are currently being held out as models, particularly in terms of abilities to test and isolate early. There could, of course, be other factors at play, such as societal cultures more responsive to government advice. But, part of the reckoning will involve asking what is it about their healthcare systems that allowed them to react so effectively. A clue might be in the usual operating capacities of such systems. For example, the UK’s NHS consistently functions at over 90% bed occupancy, giving us little room to address the peaks in required access during such crises.^13^ Similarly, in British Columbia, the western most Province in Canada, some 15 000 surgeries were cancelled over a four-week period from mid-March to mid-April to clear the way for the anticipated COVID-19 surge (which has yet to arrive). Germany spends more on its publicly-funded healthcare system than most other countries of the world, frequently operating at around 80% bed occupancy.^14^ Did this, somehow, give Germany the capacity to marshal the right facilities at the right time to combat the virus? If so, what would be the cost of getting to this level of capacity in the UK or Canada? Not only should this be characterised simply as a ‘cost,’ but rather an investment in the form of insurance (ie, surge protection). Classic health economics methods such as cost-effectiveness analysis or cost-utility analysis can be brought to the fore here to help inform decision-making. As opposed to health vs. the economy, would such ‘investment’ in capacity have avoided the economic (and health) pain to come? Experiences of different countries should inform this, demonstrating that, rather than competing with each other, health and the economy are in fact mutually dependent.^15^ Again, from our perspective the question of system capacity is fundamentally one of trade-offs to which health economics is uniquely positioned to contribute to in terms of assessing what amount of surge protection is warranted relative to other spends of the limited resources.

As outsiders looking in, in our view the public-private mix is even more of an issue for the United States. The key, here, in a Presidential election year, is how the US public view the efforts of more-coordinated single-payer systems in dealing with the virus. Despite caveats about demographics, different stages of progression of COVID-19 across countries and their different cultures, it would seem that not only the ultra-interventionist state of China but also the freer societies with single-payer health systems mentioned above have been better able to organise testing, isolation and treatment as well as behavioural initiatives required to fight coronavirus. In the United States, the public might well be asking if their own system somehow lacks the ability to organise in such similar ways, despite being, by far, the highest spender on health services in the world. Ironically, the call of Governor Andrew Cuomo of New York on March 31, 2020 to bring it all together,^16^ revealed quite blatantly that the word ‘system’ is a misnomer for US healthcare. It comprises several systems encompassing private insurance, the Veterans Administration, Medicare and Medicaid, amongst others, much of which operates without integration with primary care or public health which may have contributed to unfolding disaster in New York City. Might this now be the time? Has coronavirus shown the US population another advantage of predominantly publicly-run healthcare systems that could be the final push that is required to move to universal healthcare under a single-payer?

### Access to Testing and Ventilation: Healthcare Trade-Offs

A final area to consider and on familiar grounds to health economists are issues related to access to testing and ventilation, where, despite obvious resource limitations, decisions do not seem to have been informed by economic appraisals. One would hope that, although not made explicit to the public, multi-layered cost-benefit calculations are going on in the policy background. Hence the priority we might give to symptomatic patients, healthcare and other frontline workers testing is expanded. But, with 1m workers in the UK’s NHS alone, testing would take several months to complete and, with reverse transcription polymerase chain reaction (RT*-*PCR) telling us only whether the virus is currently present, require repeat tests and all the laboratory resources that go with such testing. Surely analyses of incremental costs and health benefits of different roll-out strategies of testing would help maximise the benefit of this (obviously) scarce resource. The same applies to the additional antibody tests which can tell whether a person has been infected in the past, but, apparently, and perhaps dangerously, with varying degrees of reliability.^17^ Careful analyses of trade-offs are required to ensure best use of this valuable resource, all in the name of ensuring gains in technical efficiency.

More drastically, issues about access to ventilation are beginning to emerge.^18^ Of course, governments are acting as fast as they can to ensure that ventilator capacity will be able to meet all the needs that may arise. Although, this may reflect public preferences for the ‘rule of rescue,’^19^ the heroic Nightingale and Louisa Jordan Hospitals in England and Scotland respectively may just be that, and, potentially, carry opportunity costs (ie, sacrifices) greater than benefits through diverting resources from social care and from other hospitals crying out for equipment, and even staff, in the very same cities. Difficult – and hopefully only occasional – choices of a cost-benefit nature will also arise, literally, at the bedside. Clinicians may be faced with deciding whether a young person with a decent chance of survival should take precedent over an older person who is severely ill and unlikely to live anyway. For those, we would want to rely on, and support, the experts – the clinicians. Speaking as health economists, we know we would. Likely, some level of ethical guidance is also required. Both economics and ethics would also seem to apply as issues of trading-off COVID-19 activities against non-COVID-19 treatments and procedures^20^ – sometimes for quite serious conditions – begin to emerge. Indeed tried-and-tested, comprehensive frameworks for resource allocation, drawing on both ethics and economics, exist and could be playing a part.^21^

## Conclusion

The issues outlined above range from broad economic considerations to really quite specific trade-offs in ensuring maximum benefit for the population and best use of publicly-funded resources. In all of this, health economic methods can make a major contribution, acknowledging of course health economics does not have all the answers. But there is a perspective and way of thinking outlined here that can be valuable when planning and seeking to make the best of this dynamic situation for society as a whole. Whether a matter of putting ourselves out there, being invited in or a combination of the two, greater visibility of health economics during the COVID-19 crisis will help, not just through the analyses provided but also in informing the major debates about allocation of society’s valuable resources that continue to ensue. That, likely more than anything, is the main reason why we need to stop asking ‘where has all the economics gone?’

## Ethical issues

Not applicable.

## Competing interests

Authors declare that they have no competing interests.

## Authors’ contributions

Both authors contributed equally to the conception and writing of the material presented.

## Authors’ affiliations

^1^Yunus Centre for Social Business & Health, Glasgow Caledonian University, Glasgow, UK. ^2^Centre for Clinical Epidemiology and Evaluation, Vancouver Coastal Health Research Institute, University of British Columbia, Vancouver, BC, Canada.

## References

[1] Twitter feed of Professor Trisha Greenhalgh @trishgreenhalgh, May 2, 2020 onwards.

[2] Holmdahl I, Buckee C (2020). Wrong but useful – what Covid-19 epidemiologic models can and cannot tell us. N Engl J Med.

[3] Scientific Advisory Group for Emergencies (SAGE): Coronavirus (COVID-19) response. https://www.gov.uk/government/groups/scientific-advisory-group-for-emergencies-sage-coronavirus-covid-19-response.

[4] Scottish Government COVID-19 Advisory Group. https://www.gov.scot/groups/scottish-government-covid-19-advisory-group/.

[5] Conseil scientifique Covid-19 (France). https://fr.wikipedia.org/wiki/Conseil_scientifique_Covid-19#Composition.

[6] Online la composizione del Comitato tecnico scientifico (Italy). http://www.salute.gov.it/portale/news/p3_2_1_1_1.jsp?lingua=italiano&menu=notizie&p=dalministero&id=4544.

[7] Netherlands Outbreak Management Team. https://www.rivm.nl/en/novel-coronavirus-covid-19/omt.

[8] Constituido el Comité Científico Técnico COVID-19 (Spain). https://www.geriatricarea.com/2020/03/23/constituido-el-comite-cientifico-tecnico-covid-19/.

[9] Karnon J (2020). A simple decision analysis of a mandatory lockdown response to the COVID-19 pandemic. Appl Health Econ Health Policy.

[10] Baker R, Donaldson C, Mason H, Jones-Lee M (2014). Willingness to Pay for Health. Encylopedia of Health Economics.

[11] Donaldson C, Baker R, Mason H (2011). The social value of a QALY: raising the bar or barring the raise?. BMC Health Serv Res.

[12] Mossialos E, McGuire A, Anderson M, Pitchforth E, James A, Horton R (2018). The future of the NHS: no longer the envy of the world?. Lancet.

[13] National Institute for Health & Care Excellence. Chapter 39: Bed Occupancy. NICE Guideline 94; March 2018.

[14] Koptyug E. Bed capacity utilization in hospitals in Germany from 1998 to 2018. https://www.statista.com/statistics/578484/bed-capacity-utilization-hospitals-germany/. Published June 8, 2020.

[15] Ratcliffe J. Why it’s not health vs the economy in the COVID-19 crisis. Committee for Economic Development of Australia website. https://www.ceda.com.au/Digital-hub/Blogs/CEDA-Blog/March-2020/Why-it-is-not-health-vs-the-economy-in-the-COVID-19-crisis. Published March 30, 2020.

[16] Barkan R. Cuomo helped New York get into this mess. The Nation. March 30, 2020. https://www.thenation.com/article/politics/covid-ny-hospital-medicaid/.

[17] Edwards A. COVID-19 tests: how they work and what’s in development. The Conversation. March 24, 2020. https://theconversation.com/covid-19-tests-how-they-work-and-whats-in-development-134479.

[18] Marsh S, Book R. Hertfordshire hospital forced to consider who should be refused oxygen. The Guardian. April 5, 2020. https://www.theguardian.com/world/2020/apr/05/hertfordshire-hospital-forced-to-consider-who-should-be-refused-oxygen.

[19] Hadorn DC (1991). Setting health care priorities in Oregon: cost-effectiveness meets the rule of rescue. JAMA.

[20] Cancer patients in limbo as treatment and surgery is cut back to cope with coronavirus pressure. ITV News. March 26, 2020. https://www.itv.com/news/2020-03-26/coronavirus-outbreak-cancer-treatment-surgery-cut-back-surge-in-covid-19-patients/.

[21] Peacock S, Ruta D, Mitton C, Donaldson C, Bate A, Murtagh M (2006). Using economics for pragmatic and ethical priority setting: two checklists for doctors and managers. BMJ.

